# Indican in the Urine of Typhoid Patients

**Published:** 1880-12

**Authors:** 


Indican in the Urine of Typhoid Patients. — A patient under the care of M. Hardy (*La Praticien,*) was attacked with fever and diarrhœa, which suggested typhoid, however, the fever soon ceased, the temperance fell to normal, and it was supposed to have been only a synocha. There still remained, however, a certain amount of swelling of the spleen and of diarrhœa which tended to support the first diagnosis. About the fourteenth day there appeared some spots which were only discovered with difficulty. In order to find a new element of diagnosis, M. Hardy examined the urine of the patient for indican, the blue matter which sometimes exists there.

To discover this a few grammes of urine should be put into a test tube, three times its volume of hydrochloric acid added, and then slightly warmed. The mixture must then be cooled, and a few grammes of ether added. The cooling is necessary on account of the great volatility of the ether; if it is put into warm liquid an explosion may occur. The mixture of urine and ether should now be agitated by closing the mouth of the tube and inverting it a few times. The ether being lighter than water will separate and collect at the top, and if indican is present, as was the case in M. Hardy's patient, there will be found a perfect characteristic blue ring. This presence of indican in the urine, which is very easy to discover, is not, however, a pathognomonic sign; it may be present in the urine of those suffering from other affections or even in good health, but it is a useful sign when added to those furnished by other symptoms.—*Med. Press and Cir.*

